# Progress in Medicine

**Published:** 1895-05-18

**Authors:** 


### PROGRESS IN MEDICINE.

### RHEUMATIC AFFECTIONS.

**Polymyositis.**—Gouget[1] writes upon eighteen recorded cases of this affection, in which he considers the diagnosis well established. The affection appears to be a severe one, for of those eighteen cases ten died. The disease generally commences insidiously with general malaise and muscular pains increased by movement and pressure. Some œdema occurs in the painful regions, and the affected muscles become swollen and distinct. Rashes of various kinds generally make their appearance. They may be erysipeloid, roseolous, urticarial, or purpuric. Fever is present, and profuse sweating is common. The disease appears to have occurred at all ages, from eight to seventy. More men have been attacked than women. The disease has to be distinguished from trichinosis and multiple neuritis. In trichinosis the disease is generally ushered in by pronounced gastro-intestinal symptoms. More muscles are generally attacked than in polymyositis, and it only occurs in those who have eaten insufficiently cooked pork. From multiple neuritis polymyositis differs in being unaccompanied by tenderness of the nerves, in the absence of affection of skin sensation, in the swelling being greater, and the symmetry less marked.

**Epidemic Muscular Rheumatism.**—Dr. Isaac Newton sends a communication to the *British Medical Journal* upon a curious febrile affection associated with severe muscular pains. The intercostal and lower abdominal muscles were first affected, rendering breathing difficult. The pain afterwards spread to the lumbar muscles. The temperature was raised from 100 to 104, but generally fell again to normal on the second or third day. The pulse was very slightly quickened. Dr. Newton looks upon the disease as distinct from influenza. Although several individuals were attacked during the space of five weeks he was unable to note facts that pointed to the disease having been infectious.

Mr. Grindon, of Oldney, stated the following week that he had seen 80 cases suffering from the symptoms described by Dr. Newton that occurred in young adults under 40. Mr. Grindon, in spite of the large number of persons attacked, did not consider the disease infectious. Dr. Donovan (Edrington) and Dr. McHardy (Cullen, N.B.) also wrote to say that similar epidemics had passed under their notice, and Dr. McHardy agreed with Dr. Newton that the disease did not appear to be infectious. Dr. McHardy had seen forty-eight cases, and in four erythematous rashes had appeared.

**Acute Torticollis and Lumbago of Articular Origin.**—Robin and Londe[3], of Paris, draw attention to the fact that rheumatic torticollis and lumbago are probably not due to affections of the muscles, as is generally supposed, but to rheumatism of the articulations of the vertebræ. The positions of greatest pain on pressure are situated over the articulations, and the writer remarks that Chomel has already drawn attention to these inter-spinal and inter-vertebral sore spots of rheumatic lumbago. One side of the spine may be affected more than the other, and the head is then deviated from the middle in order to assume the position of greatest comfort. Robin and Londe consider that the disease is best treated by jaborandi; they think that salicylates frequently fail to be beneficial.

**Rheumatism.**—Carrien[4] takes the view that rheumatism originates in imperfect elaboration of elements serving nutrition, and that insufficient elimination of their effete products, favoured or not by certain influences, fatigue, cold, &c., gives rise to auto-intoxication and the manifestation of rheumatism. He considers that the beneficial effect of salicylate of soda is threefold. It lowers the temperature, has an analgesic effect upon the articulation, and thirdly, carries off urea, uric acid, and other waste products by the kidneys. Dr. Latham,[5] referring to the use of salicylates in a paper read before the Cambridge Medical Society, states his firm belief in the efficiency of the remedies, but thinks that certain points have to be observed in order to ensure success. He formulates seven rules, amongst which the following may be mentioned: Firstly, that the salicylate acid must be obtained from the vegetable kingdom; secondly, that it must be given without an alkali or a base; thirdly, from 40 to 80 grains should be given daily for ten days; fourthly, the patient's diet should consist of milk and farinaceous food for at least a week; and fifthly, the bowels should be freely opened daily. Dr. Latham considers calomel to be a useful adjunct. Dr. F. Moritz[6] (St. Petersburg) considers that warm baths may be advantageously combined with salicylates in the treatment of rheumatism. His method is as follows: The patient is put into a warm bath of from 38 to 41 degrees C. for about twenty minutes, after which he is wrapped in a sheet and a woollen blanket, and rapidly carried back to bed, where he is covered with two additional blankets. He is then given from 16 to 24 grains of salicylate of sodium, as well as a glass of warm punch. In a very short time there is extreme diaphoresis. The sweating process is kept up for half an hour, after which one of the limbs is uncovered and rapidly washed with a sponge soaked in cold water, then quickly dried, and covered with a light woollen cover. The procedure is repeated for all the over limbs and the chest and abdomen. Lastly, all the first wraps are removed, and a clean shirt is put on. This treatment relieves pain, and the patient goes to sleep. From 40 to 60 grains of salicylate of soda are given in the twenty-four hours for the first few days, combined with a bath every morning. Later smaller doses of salicylate are given. Dr. Richard Drews, of Hamburg,[7] agrees with other observers in the value of salophen in rheumatism. He has given the drug to fifteen cases of acute rheumatism in children, in doses of from 5 to 8 grains every two hours. The pain has generally disappeared in the first twenty-six hours, and no unpleasant symptoms have been noticed. The drug was also found useful in a case of rheumatism of the neck. M. Drappier, of Ardennes,[8] records a case in which salicylates and warm baths failed to give relief in a case of rheumatism. Pilocarpin injections were therefore tried with the result that abundant sweating was followed by relief of pain and sleep. For four days the injection was repeated with the same result, after which the pain disappeared. Various external applications have been recommended for rheumatic joints, the principal ingredient being salol or salicylic acid. Thus the *Presse Médicale* for November 3rd, 1894,[9] gives the following formula: Salicylic acid five drachms, alcohol three ounces and a half, castor oil seven ounces, chloroform seventy-five grains. Compresses saturated with the solution are to be applied to the diseased joints and then covered with some impermeable material. Phenacetin has also been used as an external application.[10] The following are formulas: Phenacetin, gr. 65; lanolin, ʒvi.; olive oil, a sufficiency, the ointment to be rubbed into the inflamed part; or phenacetin, gr. 65; alcohol, O ij., to be applied as a compress to the affected joints.

[1] Gouget, La Presse Médicale, Sept., 1894; Med. Chron., Nov. 1894. [2] Brit. Med. Jour., Sept. 27, 1894. [3] Med. Week, Nov. 2, 1894. [4] Jour. des Pract., Oct. 27, 1894; Med. Chron., Dec., 1894. [5] Lancet, Jan. 19, 1895. [6] Med. Week, Sept 7, 1894. [7] Allq. Medicin Cent. Zeit., No. 60, 1894; Lancet, Aug. 25, 1894. [8] Journ. des Sc. Méd. de Lille, Sept. 15, 1894; New York Med. Jour., Oct. 1894. [9] New York Med. Jour., Dec. 1894. [10] Jour. de Med. de Paris, April 29, 1894; Ther. Gaz., Aug. 15, 1894.

